# Respiratory Impairment and Systemic Inflammation in Cedar Asthmatics Removed from Exposure

**DOI:** 10.1371/journal.pone.0057166

**Published:** 2013-02-27

**Authors:** Chris Carlsten, Anne Dybuncio, Mandy M. Pui, Moira Chan-Yeung

**Affiliations:** Respiratory Medicine, University of British Columbia, Vancouver, British Columbia, Canada; University of Texas Health Science Center at San Antonio, United States of America

## Abstract

**Background:**

Prior research has shown that removing occupational asthmatics from exposure does not routinely lead to significant improvements in respiratory impairment. These studies were of limited duration and factors determining recovery remain obscure. Our objective was to evaluate residual respiratory impairment and associated sputum and blood biomarkers in subjects with Western red cedar asthma after exposure cessation.

**Methods:**

Subjects previously diagnosed with cedar asthma, and removed from exposure to cedar dust for at least one year, were recruited. Subjects completed a questionnaire and spirometry. PC_20_ (methacholine concentration that produces 20% fall in FEV_1_ (forced expiratory volume at 1 second)) sputum cellularity and select T_h_1/T_h_2 (T helper cells 1 and 2) cytokine concentrations in peripheral blood were determined. The asthma impairment class was determined and multivariate analyses were performed to determine its relationship with sputum cell counts and serum cytokines.

**Results:**

40 non-smoking males (mean age 62) were examined at a mean interval of 25 years from cedar asthma diagnosis and 17 years from last cedar exposure. 40% were in impairment class 2/3. On average, the PC_20_ had increased by 2.0 mg/ml; the FEV_1_ decreased by 1.5 L, with greater decrease in those with greater impairment. Higher impairment was associated with serum interferon-gamma (mean = 1.3 pg/ml in class 2/3 versus 0.62 pg/ml in class 0/1, p = 0.04), mainly due to the FEV_1_ component (correlation with interferon-gamma = −0.46, p = 0.005).

**Conclusion:**

Years after exposure cessation, patients with Western red cedar asthma have persistent airflow obstruction and respiratory impairment, associated with systemic inflammation.

## Introduction

Individuals who continue to work with red cedar dust following disease diagnosis will near-uniformly continue to have respiratory symptoms and require medications to control their asthmatic symptoms[1]. Yet even among workers diagnosed with Western red cedar asthma (WRCA) and no longer exposed to WRC, the majority fail to fully resolve related symptoms, even after several years[2]. A follow-up study of 232 patients, four years after initial diagnosis, showed that of the 136 patients who were no longer exposed to WRC, only 40% recovered fully (were asymptomatic) while the remaining 60% of subjects continued to show symptoms of asthma[3]. Patients in the asymptomatic group had a significantly shorter duration of exposure before diagnosis, suggesting that they were diagnosed at an earlier stage of the disease, but even early removal from exposure was not fully protective; other hypothesized risk factors for persistent asthma, such as race, gender, atopy, smoking, and specific IgE antibodies, were shown not to be associated with persistence[3].

The finding, that longer exposure duration is a risk for long-standing impairment but that removal from exposure only occasionally leads to complete resolution of impairment, has been seen with other occupational asthmagens such as toluene diisocyanate[4]. However, these prior studies[3], [4] observed changes at approximately 5 years after removal from exposure. Our primary goal was to see whether ongoing abstinence from exposure would itself result in incrementally improved asthma status over a longer period of time, as suggested by Perfetti et al[5] in a study extending to approximately 10 years post-exposure cessation, even though the duration of follow-up was not a significant factor in a systemic review[6]. Secondarily, given the prior data that early removal is not uniformly protective in all individuals, we wondered what other factors might influence persistence. We hypothesized that ongoing functional impairment in occupational (cedar) asthmatics would correspond with active inflammation in sputum (particularly in terms of TGF-beta[7], [8] and eosinophils[9]) and/or blood[10], [11]. Notably, our study serves as a response to a recent call from an expert panel regarding outstanding questions related to occupational asthma, i.e. “besides duration of exposure…what are the other determinants of the persistence of asthma after removal from exposure?”[12]. Preliminary results from this study have been previously presented in the form of an abstract[13].

## Materials and Methods

### Ethics statement

According to the protocol approved by the ethics board at the University of British Columbia, we attempted to contact never-smoking individuals previously diagnosed with Western red cedar asthma by specific inhalational challenge and last known to be living in the lower mainland of British Columbia. Written consent was given by all participants.

### Methods

#### Peripheral blood

Peripheral blood was drawn by standard venipuncture procedure.

#### Lung function

Spirometry was carried out according to ATS criteria[14].

A methacholine challenge test was carried out according to the method of Cockroft et al[15] to determine the PC_20_. Those whose FEV1 did not fall 20% even with the 16 mg/ml dose of methacholine were assigned a PC_20_ of 16. An interviewer trained in the language of the subject's choice (English or Punjabi) administered a questionnaire modified from that used in the 2003-4 study[16] to assess recent asthma-related medications.

#### Impairment rating

The respiratory impairment rating for each subject was determined according to ATS guidelines[17]. The sum of the scores for post-bronchodilator FEV_1_ (0–4 scale), NSBH (0–3 scale), and the medications used to control asthma (0–4 scale) lead to the class of impairment, expressed as Class 0 (total score 0), class 1 (total score 1 to 3), Class 2 (total score 4 to 6), Class 3 (total score 7 to 9) and Class 4 (total score 10 to 11) and Class 5 (asthma not controlled despite maximum treatment).

#### Sputum induction, processing and cellular analysis

Sputum was induced using an aerosol of inhaled hypertonic saline by a modification of the method of Pin *et al.* [18]. Prior to sputum induction, inhaled salbutamol (200 µg) was given to inhibit possible airway constriction. FEV_1_ and FVC were measured three times before sputum induction. The best FEV_1_ value was used as the baseline. The Fisoneb ultrasonic nebulizer (Clement Clarke International Ltd.) with an output of 0.87 ml/min and an aerodynamic mass median diameter of 5.58 µm was used to deliver saline. Concentrations of saline at 3.00, 4.00 and 5.00% (each for 7 min) were given by inhalation through a mouth piece without a valve or noseclip. After each 7 min inhalation period, spirometry was repeated. The subject was asked to rinse his mouth with water and blow his nose (to reduce contamination of the sputum specimen with saliva and postnasal drip) before coughing the sputum sample into a sterile container. If the FEV_1_ fell by >10% from the baseline the same concentration of saline was used for the next inhalation. If the FEV_1_ fell by >20%, the procedure was discontinued and salbutamol given.

Sputum was analyzed within 2 hours of sample collection using the method described by Pizzichini et al[19]. The sample of sputum was transferred to a Petri dish and all visible sputum plugs were selected, placed in a pre-weighed conical tube and weighed. Freshly prepared dithiothreitol (DTT; dilution of 1 part to 9 parts distilled water) was added in a volume (ml) equal to 4 times the weight of sputum (mg) in order to homogenize the sample. The sample was vortexed for 15 seconds and then rocked using a bench rocker for 15 minutes. An equal volume of Dulbecco's phosphate-buffered saline was added and the sample was then filtered to remove particulate matter. The resultant samples were each centrifuged at 500×G for 10 minutes and the supernatant was aspirated into aliquots and stored at −80°C until use. The remaining pellet after centrifugation was resuspended in phosphate-buffered saline and the cell count was determined as follows. The number of cells per ml of processed sputum was calculated. The viability of cells was evaluated using the trypan blue exclusion method. The cell suspension was adjusted to 1×10^6^ cells/ml, and two cytospins were prepared with 50 ul of the cell suspension. These slides were air-dried, fixed and stained with Wright's stain. At least 400 non-squamous cells were counted and a differential cell count was made. The supernatant was used for cytokine analyses, as described below.

#### Serum and sputum cytokine analysis

Concentrations of the cytokines in serum (IFN-gamma, IL-10, IL-12, IL-5, TGF-beta) and sputum (IL-5, TGF-beta) were determined using MesoScaleDiscovery (Gaithersburg, MD) with limits of detection reported as 0.68 pg/mL (IFN-gamma), 0.14 pg/mL (IL-10), 0.50 pg/mL (IL-12), 0.23 pg/mL (IL-5), and 0.064 pg/mL (TGF-beta).

### Analysis

Comparison to a prior study population[16] was made, using t-tests, in terms of key demographic characteristics for those individuals with common data.

The main outcome measure, impairment, was defined by ATS criteria-based impairment classes[17], incorporating scores for lung function, NSBH, and asthma medications and then classifying in a binary fashion (Class 0/1 vs Class 2/3; no subjects were in classes 4 or 5).

The risk of having a higher impairment class (IC) based on demographic characteristics and lung function was determined by multivariate logistic regression. The risk of having a higher impairment class (IC) based on elevated sputum or serum cytokines (dichotomized at the median) was determined by multivariate logistic regression.

## Results

From our database, there were 46 individuals who met the aforementioned criteria for contact attempt who remained living and were physically able to make the trip to our study centre. Of these, 40 never-smoking male cedar asthmatics successfully completed the study; of these 65% were Caucasian, 41% were atopic, and 40% were currently on inhaled steroids. 6 subjects started the study but did not complete one of the essential study components (methacholine challenge incomplete in one subject; 5 subjects unable to produce sputum or blood).

As noted in Table 1, this population was of late middle age (mean age 62), distant temporally from initial diagnosis (25 years prior, on average) and last cedar dust exposure (17 years prior, on average). Compared to another, larger, population of individuals with cedar asthma studied by our group[16], subjects in the present study were not significantly different in terms of age, time from initial diagnosis, or percent predicted FEV_1_. However, the average PC_20_-FEV_1_ (methacholine, mg/ml) in the present study, 6.8 (range 0.1–16.0), was significantly higher than that in our prior study[16].

**Table 1:** Demographics and lung function (baseline and change from time of initial diagnosis with Western red cedar asthma).

	N	Mean	SD	Range
Age (years)	40	62.0	10.0	33, 82
Years from initial diagnosis	40	24.5	6.8	6, 38
Years from last exposure	40	17.0	9.3	1, 34
FEV_1_, percent predicted	40	83.2	16.1	48, 128
PC_20_-FEV_1_ (methacholine, mg/ml)	40	6.8	6.8	0.1,16.0
Change of PC_20_ (mg/ml)	30*	2.0	8.7	−24.1,15.0
Change of FEV_1_ (L)	40	−1.5	0.6	−2.6, −0.4
Change of FEV_1_ per year (L)	40	−0.07	0.05	−0.3, −0.01

* 10 subjects did not have PC_20_ available from the time of initial diagnosis with Western red cedar asthma

As noted in Table 2, FEV_1_ percent predicted and PC_20_ were each lower in those with moderate impairment relative to those with mild impairment (respectively: 71 v 92%, p = 0.005; 3.4 v 9.1 mg/ml, p = 0.014); the loss of FEV_1_ per year was greater in those with moderate impairment relative to those with mild impairment (80 v 60 ml, p = 0.04). In the case of FEV1, the change since the time of diagnosis was also greater in those with moderate impairment relative to those with mild impairment (−1.8 v −1.2 L, p = 0.008) and there was a similar trend in the change in PC_20_ (−1.6 v 4.0 mg/ml, p = 0.09). Years from initial diagnosis showed a trend to being fewer in those with moderate versus mild impairment (26 v 23 years, p = 0.06).

**Table 2:** Odds of increased respiratory impairment given various subject characteristics (race, atopic status and inhaled steroid use [n (%)]; age and lung function [mean (SD)]).

	IC 0/1n = 24	IC 2/3n = 16	OR*	95% CI
Race, Caucasian	15 (63)	11 (69)	1.32	0.35, 5.05
Atopy, positive	6 (32)	7 (53.8)	2.53	0.59, 10.86
Age (years)	61.8 (11.5)	61.3 (7.7)	1.00	0.94, 1.10
*Inhaled steroid use*	7 (29.2)	9 (56.3)	3.12	0.83, 11.72
*FEV_1_ percent predicted*	91.6 (12. 6)	70.5 (12.0)	0.82**	0.71, 0.94
*PC_20_-FEV_1_ (methacholine, mg/ml)*	9.1 (6.9)	3.4 (5.1)	0.86**	0.76, 0.97
Change of FEV_1_ (L)	−1.2 (0.5)	−1.8 (0.5)	0.06**	0.01, 0.49
Change of PC_20_-FEV_1_ (mg/ml)	4.0 (8.0)	−1.6 (9.1)	0.90	0.81, 1.02
Years from initial diagnosis	25.8 (6.4)	22. 6 (7.2)	0.87	0.76, 1.01
Years from last exposure	17.1 (8.3)	16.8 (10.9)	1.00	0.93, 1.07
Change of FEV_1_ per year (L)	−.06 (.05)	−0.08 (0.04)	0.005**	0.00, 0.55

*Italics* depicts the 3 components of the impairment score.

* Odds of being in IC 2/3 versus IC 0/1 with given characteristic, from logistic regression model with, in each analysis, adjustment for age (except for fevpp, where age is already incorporated, and for race, atopy, inhaled steroids) and years since last exposure (except for race, atopy, inhaled steroids).

** p<0.05

**Figure 1:**
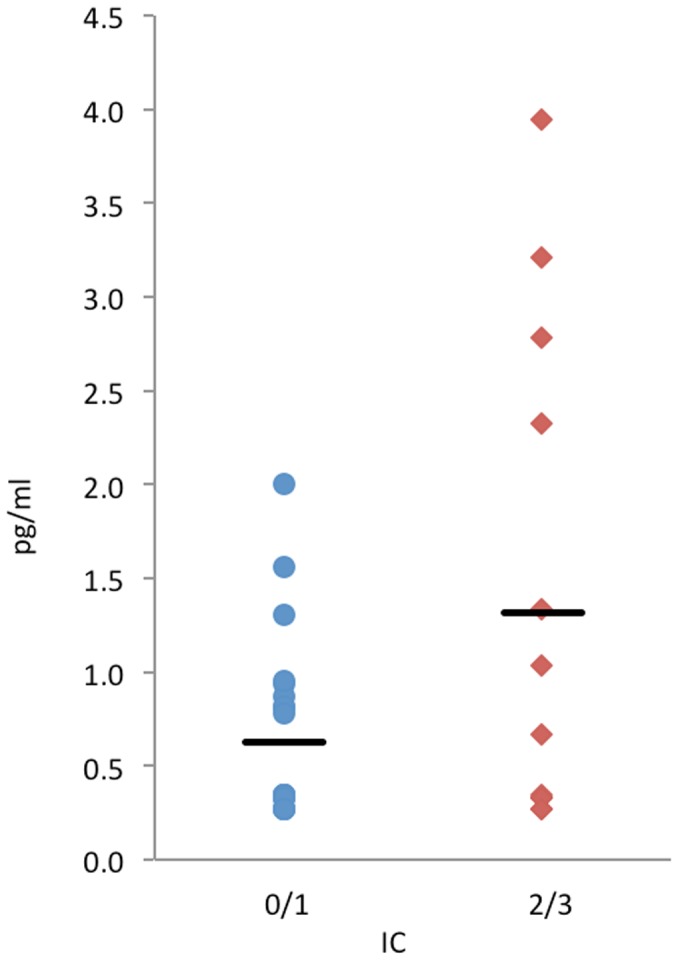
Serum interferon-gamma, stratified by higher versus lower respiratory impairment (IC  =  impairment class).

As noted in Table 3 and Figure 1, asthma-related impairment was associated with higher interferon-gamma levels in serum (1.32 pg/ml for IC2/3 versus 0.62 pg/ml for IC0/1; p = 0.04). Other cytokines in sputum and blood were not significantly associated with IC or any of its components and inference based on trends was limited given the large variability in several of these measures. There was a notable trend, amongst those with moderate impairment versus those with mild impairment, towards higher sputum eosinophilia, both in absolute terms and as a percentage of total viable sputum cells (1.4% for IC2/3 versus 0.2% for IC0/1).

**Table 3:** Serum and sputum cytokines and sputum cell counts, stratified by impairment status.

			Total	IC 0/1	IC 2/3		
			Mean (SD)	Mean (SD)	Mean (SD)	OR*	95%CI
Serum (pg/ml)		IFN-gamma	0.87 (0.9)	0.62 (0.5)	1.32 (1.3)	2.95**	1.06, 8.22
		IL-10	4.98 (17.2)	2.40 (4.6)	9.54 (28.1)	1.03	0.96, 1.10
		IL-12	6.23 (21.7)	3.21 (8.1)	11.58 (34.7)	1.02	0.98, 1.06
		IL-5	0.84 (1.2)	0.84 (1.2)	0.83 (1.4)	0.95	0.53, 1.71
		TGF-beta	2048.04 (1145.0)	2078.18 (1294.2)	1974.86 (741.5)	1	1.00, 1.00
Sputum	ng/ml supernatant	IL-5	0.07 (0.1)	0.07 (0.1)	0.06 (0.06)	0.23	0.0, 778.61
		TGF-beta	1491.17 (1012.5)	1506.35 (1150.4)	1460.80 (712.7)	1	1.00, 1.00
	Cell counts	Eosinophils	2.40 (7.7)	0.68 (1.7)	5.36 (12.3)	1.16	0.89, 1.51
		% Eosinophils	0.60 (1.9)	0.17 (0.4)	1.34 (3.1)	1.8	0.62, 5.28
		Neutrophils	298.53 (68.7)	287.84 (75.1)	317.00 (54.1)	1.01	1.00, 1.02
		% Neutrophils	74.85 (17.2)	72.42 (18.9)	79.06 (13.3)	1.03	0.98, 1.08

* Odds of being in IC 2/3 versus IC 0/1 from logistic regression model appropriate to each variable, each adjusted for age and years since last exposure.

** p = 0.04.

Given the literature suggesting the importance of TGF-beta as a mediator of airway remodeling leading to airway reactivity, *post hoc* analyses were performed to assess the relationship between serum TGF-beta and PC_20_. Those whose PC_20_ had decreased, from the time of diagnosis to the time of the current reassessment, had a significantly higher serum TGF-beta than those whose PC_20_ had increased over that interval (3.1 vs 1.4 pg/ml, respectively [p = 0.05]; p = 0.02 using linear regression of continuous PC_20_, controlled for age)

## Discussion

Prior work from our group[16] demonstrated that subjects no longer working due to problems associated with asthma had the highest prevalence of chronic and acute asthma-like respiratory symptoms and a reduced quality of life compared to those who continued to work. However, these patients did not undergo lung function and laboratory testing to objectively evaluate asthma severity. In our current study, we evaluated such objective measures in order to better understand factors associated with persistent asthma in those with WRCA but removed from exposure. Our work is particularly important because it represents *the longest follow-up of occupational asthma, to our knowledge, in the literature*; given the mean follow-up of 25 years since diagnosis and 17 years since removal from exposure, the size of our reported cohort is considerable. Another notable distinction of our work is that we excluded all smokers. Other follow-up studies of occupational asthma, including our own[3], [20] typically have included smokers and attempted to account statistically for the potential confounding effect of smoking. However, we excluded them given the strong tendency for such exposure to confound, in spite of efforts to control statistically, analyses intended to focus on other exposures related to obstructive lung disease.

Neither sputum eosinophils nor neutrophils were significantly associated with impairment in our study. This was somewhat surprising since, in a prior study of patients with WRCA who were removed from WRC dust for a mean of approximately 10 years[21], we found a significant correlation between the percentage of eosinophils in sputum and the degree of respiratory impairment/disability using the same ATS impairment rating, providing quantitative evidence to support the rating, and we noted similar findings by Maghni and colleagues[20] and Lemiere and colleagues[22]. Our results may suggest that airway inflammation may decrease and become less influential on impairment with prolonged time away from exposure (as is the case in the present study). However, each of eosinophils and neutrophils trended towards association with impairment, suggesting that perhaps with a larger sample size we may have shown an association similar to that of Maghni et al[20] whose sample size was considerably larger than ours.

It is revealing to compare the improvement in non-specific airway responsiveness in our study, covering an average of 17 years since removal from exposure, to that from prior studies. We found a mean improvement in PC_20_-FEV_1_ of 2.0 mg/ml methacholine. Interestingly, this is greater than the improvement noted by Lemiere and colleagues[22] over just 4 years post-exposure cessation, but similar to the 2.2 mg/ml improvement noted by Perfetti et al[5] with an average of 6 years since diagnosis. Our finding of elevated interferon-gamma is consistent with proposed pathogenesis of asthma persistence as proposed by Panetteiri et al[23] and with the findings of the Normative Aging Study[24], in which interferon-gamma was associated with lung function decline. Furthermore, that serum interferon gamma was associated with asthma-related impairment in our study expands on a previous study that found significant association between airway responsiveness and serum interferon gamma[25].

We are limited by not having data on potential coincident cardiovascular disease in our subjects; though it seems unlikely that such non-respiratory disease would confound the association between asthma-related impairment and interferon-gamma, the possibility of such confounding exists given the likely association between chronic respiratory disease and cardiac disease[26]. Conversely, one wonders if ongoing inflammation may put such individuals at risk for future conditions, such as atherosclerosis, associated with systemic inflammation.

In any case, our data supports the consensus that minimizing exposure both in terms of exposure intensity and duration remains critical and that it is naïve to expect that time alone will ‘cure’ the disease; while our subjects on average improved in terms of impairment and steroid usage, approximately two-thirds still had a positive methacholine challenge test and reduced HRQL scores. Thus, it remains imperative to provide effective, long-term and individualized asthma management strategies for WRCA patients removed from exposure. For many WRCA patients, even after a long time removed from exposure, reducing AHR by appropriate pharmacology should remain central to asthma management and yet our study suggests that the use of inhaled corticosteroids diminishes over time in spite of ongoing AHR. Care providers should stringently resist this trend and compensation systems should continue to pay for such medications so long as AHR persists. Studies have shown that patients with occupational asthma who left work suffered considerable economic hardship. The income of many patients was reduced by at least half if they were unable to find alternative employment. Understanding the dynamics of asthma persistence and severity in those with WRCA will ultimately help us better control this disease and also minimize the financial impact of WRCA.

In summary, we found that significant asthma-related respiratory impairment persists in 40% of cedar asthmatic even long since (on average, 17 years) removal from exposure and is associated with a classical marker of circulating inflammation (interferon-gamma). This suggests that even many years after exposure cessation, non-specific inflammation persists in the blood of impaired individuals.
